# Molecular Cloning of *phd1* and Comparative Analysis of *phd1, 2*, and *3* Expression in *Xenopus laevis*

**DOI:** 10.1100/2012/689287

**Published:** 2012-05-03

**Authors:** Dandan Han, Luan Wen, Yonglong Chen

**Affiliations:** ^1^Key Laboratory of Regenerative Biology, Guangzhou Institutes of Biomedicine and Health, Chinese Academy of Sciences, 510530 Guangzhou, China; ^2^Graduate University of Chinese Academy of Sciences, 100049 Beijing, China; ^3^Section on Molecular Morphogenesis, Laboratory of Gene Regulation and Development, Program on Cell Regulation and Metabolism, Eunice Kennedy Shriver, National Institute Child Health and Human Development (NICHD), National Institutes of Health (NIH), Building 18T, Room 106, 18 Library DR MSC 5431, Bethesda, MD 20892-5431, USA

## Abstract

Intensive gene targeting studies in mice have revealed that prolyl hydroxylase domain proteins (PHDs) play important roles in murine embryonic development; however, the expression patterns and function of these genes during embryogenesis of other vertebrates remain largely unknown. Here we report the molecular cloning of *phd1* and systematic analysis of *phd1*, *phd2*, and *phd3* expression in embryos as well as adult tissues of *Xenopus laevis*. All three *phds* are maternally provided during *Xenopus* early development. The spatial expression patterns of *phds* genes in *Xenopus* embryos appear to define a distinct synexpression group. Frog *phd2* and *phd3* showed complementary expression in adult tissues with *phd2* transcription levels being high in the eye, brain, and intestine, but low in the liver, pancreas, and kidney. On the contrary, expression levels of *phd3* are high in the liver, pancreas, and kidney, but low in the eye, brain, and intestine. All three *phds* are highly expressed in testes, ovary, gall bladder, and spleen. Among three *phds*, *phd3* showed strongest expression in heart.

## 1. Introduction

Aerobic organisms in response to inadequate oxygen availability evolved sophisticated systems to adapt the environment, in which hypoxia-inducible factors (HIFs) play pivotal roles [1–3]. HIF functions as a heterodimer consisting of an unstable alpha subunit, such as HIF1*α* or HIF2*α*, and a stable beta subunit, such as HIF1*β*, also called ARNT1. Under normoxic conditions, the constitutively expressed alpha subunits are hydroxylated by prolyl hydroxylase domain containing proteins, such as PHDs and FIH, whose activity is absolutely dependent on oxygen. The hydroxylation generates binding sites for the von Hippel-Lindau (pVHL) tumor suppressor protein, a component of a ubiquitin ligase complex. Consequently, the alpha subunits are polyubiquitinated and subjected to proteasomal degradation [1, 3]. In contrast, under hypoxic conditions, the activity of PHD proteins is compromised due to low oxygen level and HIF alpha subunits are stabilized, which form active heterodimers with HIF1*β* to transcriptionally activate 100–200 genes, including genes involved in erythropoiesis, angiogenesis, autophagy, and energy metabolism [3].

The PHD proteins belong to an Fe(II) and 2-oxoglutarate-dependent oxygenase superfamily. There is only a single PHD family member called Egl9 in worm *Caenorhabditis elegans* and in the fly *Drosophila melanogaster*, while higher metazoans like the vertebrates contain three PHD genes [2, 3]. Although *egl9-*mutant worms are viable [4, 5], inactivation of *egl9 *in drosophila and *Phd2 *in mice, respectively, both resulted in embryonic lethality [6, 7]. It is intriguing to investigate if deletion of any *phd *genes could cause a lethal phenotype in other vertebrate organisms. *Phd1^−/−^* and *Phd3^−/−^* mice were normal [7]; however, sophisticated compound and conditional knockout of *Phd1, 2, *and *3 *in mice has revealed an important oxygen sensing function of PHDs in angiogenesis [8, 9], erythropoiesis [10–12], and cardiogenesis [7, 13, 14]. The tissue- or cell-type-specific functions of Phds defined in mice are well correlated with their abundant expression in corresponding tissues and cells. Except for an early report on the characterization of the temporal mRNA expression profile of *phd2 *and *phd3 *in *Xenopus *[15], it appears that there are no systematic studies on *phd *genes in other vertebrate organisms. Here, we cloned the open reading frame of *phd1 *and examined the temporal and spatial expression profiles of three *phd *genes in developing *Xenopus laevis *embryos as well as in adult tissues. Our data provide a basis for further functional analysis of these genes in the frog system.

## 2. Materials and Methods

### 2.1. Cloning of *Xenopus laevis phds*

As the *Xenopus laevis phd1 *(BC159341) in GenBank database is only a partial cDNA lacking the 5′ terminal sequences, we designed the upstream primer (5′ ACTCTGATCTGCAGTAGGAGTTGAAT 3′) according to the sequence of *phd1 *locus in *Xenopus tropicalis *genome sequence and downstream primer (5′ ATCCCTGTTACACAGTACCAGGGCACGAG 3′) from the partial *phd1 *cDNA (BC159341) sequence and successfully amplified the whole open reading frame of *Xenopus laevis phd1 *by RT-PCR using *Xenopus laevis *tadpole cDNA as templates. The obtained PCR fragment was cloned into pGEMT-easy (Promega) vector, verified by sequencing, and deposited in GenBank database with accession number (GU108333.1). *Xenopus laevis phd2 *and *phd3 *cDNAs were also cloned into pGEMT-easy (Promega) by RT-PCR with the following primers designed according to their sequences in GenBank database. *phd2: *forward 5′ AATGGCTGGTGGAGGAAGCGAGGGTTCTAAC 3′ and reverse 5′ TTCTAGACTTCTTTAACAGCTGGATCAGATG 3′; *phd3*: forward 5′ TATGCCGCCAGGATCTCCCCCATTCGATTTC 3′ and reverse 5′ TCAGCTTTCTTTAGTGGGAGGCTCTTCTCTG 3′. These primers were chosen to clone less conserved regions among three *phds* and thus to reduce possible cross signals during whole mount in situ hybridization with antisense probes generated from these plasmids.

### 2.2. Embryo Manipulation

Wild-type *Xenopus laevis *eggs were obtained by injecting 1000 IU of human chorionic gonadotrophin (HCG) into the dorsal lymph sacs of adult females 6–8 hours before egg collection. Eggs were fertilized in vitro with minced testes, dejellied with 2% cysteine hydrochloride (pH 7.8–8.0) 30 minutes after fertilization, and cultured in 0.1X MBS (1.76 mM NaCl, 48 *μ*M NaHCO_3_, 20 *μ*M KCl, 200 *μ*M Hepes, 16 *μ*M Mg_2_SO_4_, 8 *μ*M CaCl_2_, 6 *μ*M Ca(NO_3_)_2_, pH 7.4) buffer. Embryos were staged according to Nieuwkoop and Faber [16].

### 2.3. RNA Extraction and RT-PCR

Freshly collected tissues were powdered with mortar in liquid nitrogen. Total RNA from embryos and powdered tissues was extracted by using Trizol (Invitrogen) according to the manufacturer's instruction and was digested with DNaseI (Roche). First strand of cDNA was synthesized using superscript I M-MLV reverse transcriptase (Invitrogen). The annealing temperatures and PCR cycle numbers (in parentheses) and the sequences of primers used in the RT-PCR reactions are as follows: *phd1*: (55°C, 28) forward 5′ CAGTCAGAGGACCATACCATC 3′ and reverse 5′ CCTTTGCATCGAAATACCAG 3′; *phd2*: (55°C, 28) forward 5′ CACGGCATCTTTGTGCTTGA 3′ and reverse 5′ GAGTCTTTGCATCCCATTGTTTAT 3′; *phd3*: (55°C, 28) forward 5′ TGCTCTGTGGCAACCGACTT 3′ and reverse 5′ CATGAGGGTTACGCCTATCAG 3′; *ornithine decarboxylase*: (55°C, 23) forward 5′ TGAATTGATGAAAGTGGCAAGG 3′ and reverse 5′ CAGGGCTGGGTTTATCACAGAT 3′.

### 2.4. Whole Mount *In Situ* Hybridization

Embryos were fixed in MEMFA (0.1 M MOPS pH 7.4, 2 mM EDTA, 3.7% Formaldehyde) for 1 hour at room temperature and stored in ethanol at −20°C. Whole-mount *in situ *hybridization was performed in principle as described by Harland [17], with modifications as reported in Hollemann et al. [18]. To generate digoxigenin-labeled antisense probes, the *phd1*/pGEMT-easy, *phd2*/pGEMT-easy, and *phd3*/pGEMT-easy plasmids were linearized with SalI and transcribed with T7 RNA polymerase.

## 3. Results

### 3.1. Isolation of *Xenopus laevis phd1*

**Figure 1:**
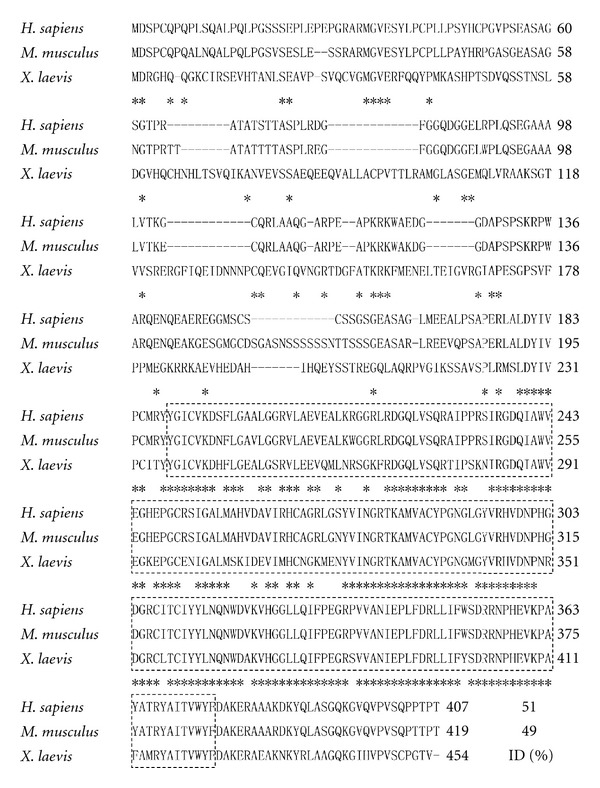
Predicted primary sequence of *Xenopus phd1* in comparison with human and mouse *Phd1*. Stars indicate identical amino acids in all three species. Hyphens represent gaps introduced for optimizing the alignment. Dashed rectangles demarcate the highly conserved prolyl 4 hydroxylase domain. ID stands for the percentage of amino acid identity of *Xenopus laevis phd1* in comparison with human and mouse *Phd1*.

**Figure 2:**
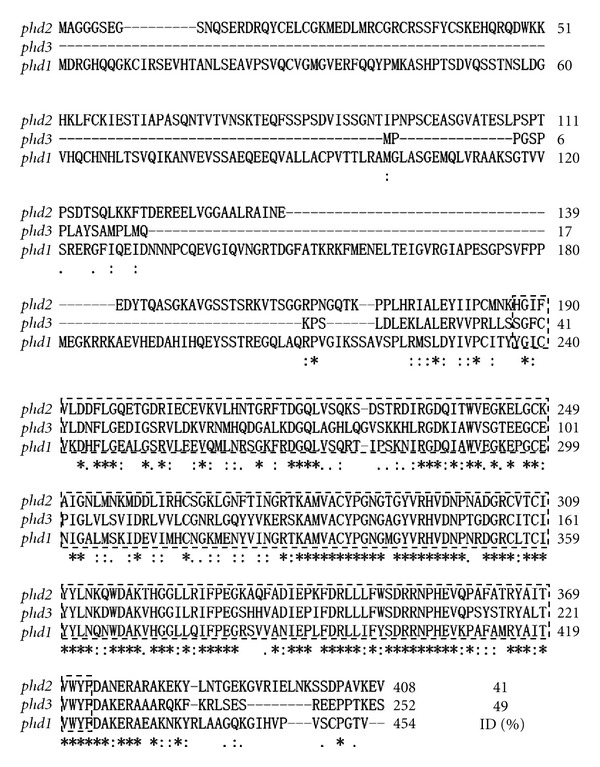
Comparison of the amino acid sequences of three *Xenopus* phds. Stars indicate identical amino acids in all three phds. Hyphens represent gaps introduced for optimizing the alignment. Dashed rectangles demarcate the highly conserved prolyl 4 hydroxylase domain. ID stands for the percentage of amino acid identity of *Xenopus laevis phd1* in comparison with *Xenopus phd2* and *phd3*.

There are three mammalian PHD genes, namely PHD1, PHD2, and PHD3 [3]. Isolation of *Xenopus laevis *homologues of PHD2 and PHD3 has been reported [15]. The amino acid sequence deduced from the whole open reading frame of *Xenopus laevis phd1 *shares 51.6% and 49.2% identity with human and mouse PHD1, respectively. Within the highly conserved prolyl 4 hydroxylase domain, the frog sequence shares 80.7% and 80.2% identity with human and mouse prolyl 4 hydroxylase domains, respectively (Figure 1). Among three *Xenopus laevis* phds, the primary amino acid sequence of phd1 shares 41% and 49% identity with those of phd2 and phd3, respectively (Figure 2).

### 3.2. Spatial and Temporal Expression Profiles of *phds*

**Figure 3:**
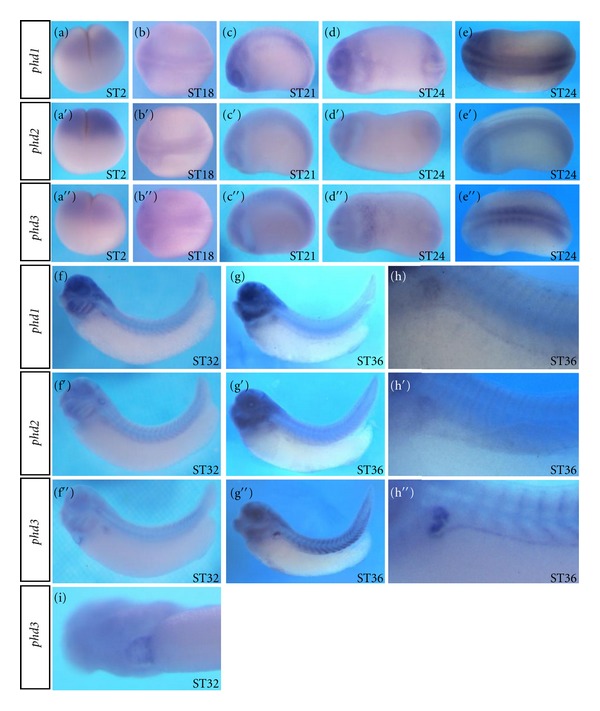
Spatial expression of *phd1, 2, *and *3 *in *Xenopus *embryos revealed by whole-mount in situ hybridization. (a–a′′) Lateral views with animal pole up. (b–b′′) Dorsal views with head towards left. (c–c′′) Lateral views with head towards left. (d–d′′) Ventral views with head towards left. (e–e′′) Dorsal views with head towards left. (f–g′′) Lateral views with head towards left. (h–h′′). Higher magnification views of (g), (g′), and (g′′), respectively. (i) Ventral view of (f′′) with head towards left.

Whole-mount in situ hybridization analyses indicate that at cleavage stages of development, higher levels of maternal transcripts for all three *phd *genes were detected in the animal hemisphere with *phd2 *showing the strongest signal (Figure 3(a), 3(a′), and 3(a′′)). At neurula stages of development, all three *phds *showed weak and relatively broad expression on the dorsal side (Figure 3(b)–3(c′′)). At early tail bud stage of development, the dorsal signals became more restricted with *phd1 *and *phd3 *expression being stronger than phd2 expression (Figures 3(e), 3(e′), and 3(e′′)). In addition, a faint signal on the anterior-ventral side of stage 24 embryos was detected for *phd1 *and *phd3 *(Figures 3(d) and 3(d′′)). At tail bud stages of development, more differential expression of all three *phds *was detected in brain, eyes, branchial arches, otic vesicle, and pronephros (Figures 3(f)–3(h′′)). A clear signal was detected for *phd3 *expression in developing heart (Figure 3(i)).

**Figure 4:**
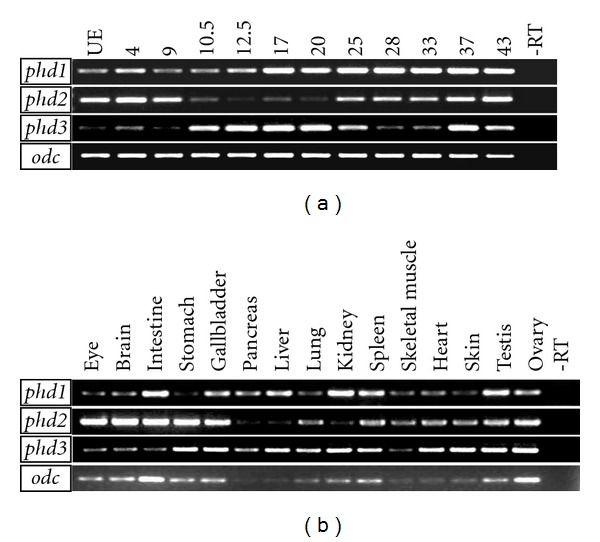
Temporal expression profile and adult tissue expression patterns of *phd1*, *2*, and *3* revealed by RT-PCR analyses. (a) Temporal expression profile of *phd1*, *2*, and *3* in *Xenopus *embryos. (b) Expression of *phds *in *Xenopus *adult tissues. *odc *was employed as a loading control. UE: unfertilized eggs.

RT-PCR analysis revealed that, up to stage 33, expression levels of *phd2 *and *phd3 *just fluctuated in a complementary manner, which has been verified by at least three times of independent experiments (Figure 4(a)). Relatively low level of *phd1 *expression maintained till gastrulation and constantly higher expression was detected from neurula stages onwards for *phd1 *(Figure 4(a)).

### 3.3. The Expression of *phds* in *Xenopus* Adult Tissues

Overall, transcripts of all three *phds *are detectable in all the adult tissues analyzed (Figure 4(b)). It is of special interest to note that *phd2 *and *phd3 *showed complementary expression in several tissues. For instance, *phd2 *is highly expressed in the eye, brain, and intestine, but low in the liver, pancreas, and kidney. On the contrary, expression levels of *phd3 *are high in the liver, pancreas, and kidney, but low in the eye, brain, and intestine. All three *phds *are abundantly expressed in testes, ovary, gall bladder, and spleen. Among three *phds*, *phd3 *showed strongest expression in heart.

## 4. Discussion

In this study, we report the isolation of the whole open reading frame of *Xenopus laevis phd1 *and characterization of the expression profiles of all three *phd3 *in *Xenopus *embryos as well as in adult tissues. Consistent with the previous report [15], we detected a complementary fluctuating temporal expression profile of *phd2 *and *phd3 *during *Xenopus *early embryogenesis. Furthermore, we found complementary expression of *phd2 *and *phd3 *in several adult tissues. In mice, several lines of evidence have indicated that PHD2 functionally coordinates with PHD3 and *Phd3 *is induced upon *Phd2 *loss [13, 14]. The functional link between phd2 and phd3 in *Xenopus *remains to be investigated.

*phd3 *expression in early *Xenopus *embryos revealed by whole-mount in situ hybridization analysis is reminiscent of zebrafish *phd3 *expression [19]. *Xenopus fih *and *hif1*α**showed similar spatial expression patterns (data not shown). Thus, in *Xenopus*, it appears that the oxygen homeostasis-related genes, *phd1, 2, 3*, *fih*, and *hif1*α**, may constitute a synexpression group. Consistent with the data in mice [20], *Xenopus phd3 *also showed highest levels of expression in adult heart. All three *phds* display expression in the pronephros. It has yet to be defined if *Xenopus *phds play specific roles in the heart and kidney development.
